# Complete Genome Sequence of Pantoea agglomerans TH81, Isolated from a Permafrost Thaw Gradient

**DOI:** 10.1128/MRA.01486-18

**Published:** 2019-01-03

**Authors:** Jennie Humphrey, Taylor Seitz, Tracie Haan, Anne-Lise Ducluzeau, Devin M. Drown

**Affiliations:** a Department of Biology and Wildlife, University of Alaska Fairbanks, Fairbanks, Alaska, USA; b Institute of Arctic Biology, University of Alaska Fairbanks, Fairbanks, Alaska, USA; University of Maryland School of Medicine

## Abstract

In this report, we describe the complete genome assembly of a Pantoea agglomerans isolate, TH81, collected from a boreal forest soil associated with permafrost thaw. Using both Nanopore and Illumina sequences, we assembled four circular contigs totaling 4,983,504 bp (*N*_50_, 4,127,869 bp), a complete chromosome with three plasmids.

## ANNOUNCEMENT

We collected soil from the Fairbanks Permafrost Experiment Station in Alaska (64.875646°N, 147.668981°W). We have been sampling soil to understand microbial community response to permafrost thaw. The isolate described in this study, Pantoea agglomerans TH81, comes from a project on the coselection of heavy-metal tolerance and antibiotic resistance.

We homogenized the active layer from two cores and added 1 g to tryptic soy broth (TSB). After 48 h at 22°C, we plated a dilution on TSB and used the streak plate method three times to purify our randomly selected isolate. From this purified isolate, we inoculated a liquid culture of TSB, incubated it at 22°C overnight, and used 1.8 ml to extract DNA using the DNeasy UltraClean microbial kit (Qiagen).

We used 1 μg of DNA to prepare a rapid sequencing library (VSK001 workflow) using the VolTRAX device (Oxford Nanopore Technologies [ONT]). We used the MinION device (ONT) for sequencing on an r9.4.1 flow cell (FLO-MIN106) for 48 h (VSK001 script). Albacore (version 2.3.1; ONT) base-called 2,404,454 passed reads, yielding 6,077,553,166 bp (average length, 2,528 bp). We used Porechop version 0.2.3 (https://github.com/rrwick/Porechop) for adapter trimming and filtered by length (≥1,000 bp) and quality (≥10 q) with Filtlong version 0.2.0 (https://github.com/rrwick/Filtlong) to yield 2,373,077,035 bp in 784,676 reads (average length, 3,024 bp). We used Filtlong to subsample 180 Mb, prioritizing by length (–length_weight 10), yielding 180,008,253 bp in 17,535 reads (average length, 10,266 bp; largest, 26,036 bp).

The Genomics Core at the University of Alaska Fairbanks prepared a Nextera XT library for sequencing on an Illumina MiSeq platform using version 3 reagents to generate 114,382 paired reads. We trimmed adapters with TrimGalore version 0.5.0 (https://github.com/FelixKrueger/TrimGalore) to yield 31,026,933 bp.

We used Unicycler version 0.4.6 (1) for hybrid assembly using both the subsampled Nanopore reads and trimmed Illumina reads. This method uses Racon version 1.3.1 (2) and Pilon version 1.22 (3) for polishing. Our 36× coverage assembly yielded 4 circular contigs totaling 4,983,504 bp (*N*_50_, 4,127,869 bp), with a 4,127,735-bp chromosome (GC content, 55.17%) along with 3 circular plasmids of 520,950 bp (GC content, 53.69%), 182,175 bp (GC content, 52.3%), and 152,526 bp (GC content, 48.87%).

Prokka version 1.12 (4) assigned 75 tRNAs, 22 rRNAs, and 4,537 coding sequences (CDS). We found 99% identity to Pantoea agglomerans (strain NCTC9381) using BLASTn (5) against the NCBI 16S rRNA database and 99% identity of the *rpoD* gene in Pantoea agglomerans (strain C410P1) in a comparison to the NCBI nonredundant database. We therefore assign this isolate as Pantoea agglomerans TH81.

### Data availability.

This whole-genome shotgun project has been deposited in GenBank under the accession numbers CP031649 to CP031652. The versions described in this paper are the first versions, CP031649.1 to CP031652.1. Raw data for this project can be found in the GenBank SRA under accession number PRJNA486198.
